# A new automatic stitching method for full-length lower limb radiography

**DOI:** 10.3389/fsurg.2022.1000074

**Published:** 2022-10-14

**Authors:** Tariq Alkhatatbeh, Jia Lin Wang, Wei Jia Zhang, Yong Wei Li, Yong Xia, Wei Wang

**Affiliations:** ^1^ Department of Bone and Joint Surgery, The Second Affiliated Hospital of Xi’an Jiaotong University, Xi’an, China; ^2^ School of Computer Science and Engineering, National Engineering Laboratory for Integrated Aero-Space-Ground-Ocean Big Data Application Technology, Northwestern Polytechnical University Youyi Campus of Northwestern Polytechnical University, Xi’an, China

**Keywords:** x-ray image stitching, feature detection, canny algorithm, full-length lower limb radiography, radiography stitching, panoramic radiography

## Abstract

Full-length lower limb x-rays are used to diagnose and plan surgical procedures, such as Total Knee Arthroplasty (TKA) and High Tibial Osteotomy (HTO). Due to the size limitation of digital radiography (DR), panoramic x-ray images cannot be obtained in a single exposure, necessitating multiple exposures and image stitching. In favor of manually constructing full-length x-ray images, we propose a new feature-based automated method for stitching together x-ray images. This new method is based on Canny algorithm, which detects and aligns bone edges before fusing them using a Wavelet form domain. Twenty-eight sets of lower limb x-ray images obtained from our hospital have been stitched and evaluated. The hip, knee, and ankle (HKA) angle was computed in two different ways then compared to manually stitched x-ray images by an expert. The stitching time was only three seconds, and the *P*-value was *P* = 0.974, and an accuracy rate of 100% was found. This method demonstrated greater precision and speed than both manually stitched x-ray images and previously published methods.

## Introduction

High Tibial Osteotomy (HTO) and Total Knee Arthroplasty (TKA) are frequent and effective surgical interventions for the treatment of osteoarthritis. Planning and evaluating these interventions requires a full-length x-ray image of the lower extremity (1). X-ray images of the lower limb consist of three parts: the hip, the knee, and the ankle. Due to the size limitation of digital radiography (DR), a full-length limb cannot be captured at once, necessitating multiple exposures that produce multiple radiographs that must be combined and fused (2). X-ray image stitching was developed to assist in joining x-ray images and displaying the desired portion in a single image. Conventional stitching generally requires an expert to independently input, align, and merge x-ray images (3), but this process may be compromised or troubled under heavy workloads (4). Therefore, researchers developed automated x-ray stitching that utilized various factors and features to achieve its purpose. Based on the method applied, automated x-ray stitching can be classified as either pixel-based or feature-based (5). Several pixel-based methods had previously been introduced; in 2013, Samsudin proposed a method that used MACE’s filter to obtain the overlapping region between two x-ray images of the hand, then stitched them together. An accuracy of 80% was found and his method required a 30 percent overlapping region for successful stitching, which was not always present (5). Similarly, Yang presented in 2016 another pixel-based method that used a phase correlation, a quick way to register x-ray images (6). This method estimated the relative translation between two x-ray images with an overlapping region and then stitched them together. It was only used to combine two x-ray images simultaneously with an accuracy rate of 90%. Therefore, their efficacy in regards to lower limb x-ray stitching cannot be demonstrated. In 2018, Ben Zekri proposed a new automated stitching method based on features using a Sobel derivative that detected bone edges and shafts to determine the overlapping region and complete the stitching. The Hip, Knee, and Ankle angle was calculated and compared to x-ray images created by an expert to evaluate his method. The results obtained were extremely promising as accuracy rate was found to be 100%, but the overall stitching time of 15 s was relatively lengthy (7). Aside from these, another method used a ruler to help align the x-ray images (8), and one more was built around a c-arm mobile x-ray device (9). And this defeats the purpose of having a dependent and reliable automated x-ray image stitching method for lower limb x-rays. Therefore, a method of precise and rapid stitching must be developed. This method could be of a significant importance economically, as it reduces the need of expensive advanced DR systems that may not be available everywhere; which reflects to more affordable and accessible options for both patients and surgeons. Besides, it could aid Orthopedic technicians with their workloads by reducing the time and effort needed for manual x-ray stitching.

The Canny algorithm is a well-known edge detector that was introduced in 1986; it can detect the edges of objects with a low error rate, discarding false edges and ensuring accurate detection (10). In this study, we propose a new Canny-based method for stitching x-ray images of the lower limb. Our method relies solely on the accurate and rapid detection of lower limb bone edges to align and proceed with the stitching. Unlike previous methods, this minimizes the need for additional equipment or materials such as rulers or external markers. We can simultaneously manipulate not only two but three x-ray images, which are required to create a full-length x-ray image of the lower limb.

## Materials and methods

### Data of patients

To perform this experiment, a total of 28 patents’ lower limb x-ray images were collected from the database of Xi’an Jiaotong University’s second affiliated hospital in JPG form. A hip, a knee, and an ankle,with each image dimension of 3408*3320 pixels, and a resolution of 150*150; and a full-length lower limb x-ray image stitched by an expert were included in each set of these x-ray radiographs.The DR system used was Sedecal “NOVA FA” that had a flat panel detector. The distance between the x-ray source and the patient was 150 CM. In total, 112 images were utilized for this study. The Second Affiliated Hospital of Xi’an Jiaotong University has granted permission for this study to collect and use patient information. This hospital’s x-ray system was a linear system in which the x-ray source moved from top to bottom to capture images.

### Data processing steps

To perform the desired x-ray stitching, the following four steps were taken: (1) Image input: hip, knee, and ankle x-ray images were selected and input. Each image was down-sampled to decrease its file size and increase its processing speed. (2) Detection of features: A Gaussian filter was initially used to smooth the images and reduce noise. This would aid in the detection of bone edges. Then, we applied the Canny Algorithm to detect the lower limb bone edges. (3) Overlapping region estimation: Based on horizontal shifting, we computed the image position transformation (x -direction offset and *y* -direction offset) to find the overlapping region. (4) Stitching of x-ray images: A Wavelet transform domain was used to stitch and merge all the images and create a full-length x-ray image.

#### Image input

The full x-ray image of the lower limb consists of three parts: the hip, knee, and ankle images. To construct image stitching successfully, we must first input these three x-ray images in the specified order. Images then were down-sampled in size to enhance the processing speed.

#### Features detection

**Figure 1:**
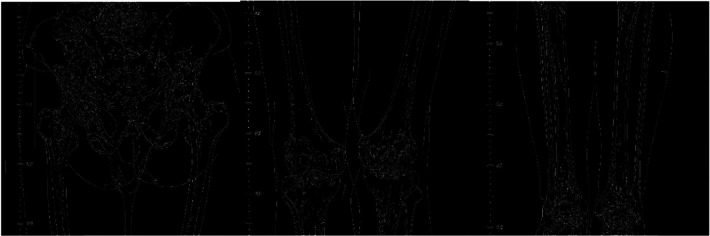
Three parts that consist the full-length lower limb x-ray image, the hip, the knee and the ankle, edges extracted by canny algorithm.

As noise can mislead any edges detection algorithm, it was essential to apply a 5*5 Gaussian filter first to remove and reduce the noise. Bone edges were then extracted using the Canny algorithm. Assuming that the original image was a two-dimensional (2D) matrix $I\in R^{H\times W}$, then $I_{x,y}$ represents the first *x* column and *y* the pixel value of the row. The edge information graph $E_{x,y}\in \{0,1\}$ indicated whether the corresponding position of the original image was an edge or not (1 is an edge, 0 for non-edge). Figure 1 shows the edges detected after applying the Canny algorithm to x-ray images.

#### Overlapping region estimation

Since the patients were required to stand still during the collection of x-ray images of the lower limbs, this study assumed that there was no rotation perspective or other transformations in the images; therefore, our method was only based on horizontal shifting, which neglects any vertical shifting. This study only needed to calculate the image position transformation (x -direction offset and *y* -direction offset) to find the overlapping region.

After extracting features using the Canny algorithm, this study calculated the distance D $D_{x,y}$ as follows:

$$D_{x,y}=\underset{x^{\prime },y^{\prime }}{\mathrm{argmin}}\sqrt{{\left(x-x^{\prime }\right)}^{2}+{\left(y-y^{\prime }\right)}^{2}},(E_{x^{\prime },y^{\prime }}=1)$$

$D_{x,y}$ represents the distance to the nearest edge (x,y).

In the matching process, this study used the overlapping region of the two images to evaluate the registration quality of the two images. The overlapping area of the two images is two equal-sized areas. If their edge map and distance map is considered as $E^{1},E^{2}$ and $D^{1},D^{2}$, then the specified matching distance A is:

$$A=\frac{\sum_{x,y}E_{x,y}^{1}D_{x,y}^{2}}{\sum_{x,y}E_{x,y}^{1}}+\frac{\sum_{x,y}E_{x,y}^{2}D_{x,y}^{1}}{\sum_{x,y}E_{x,y}^{2}}$$

**Figure 2:**
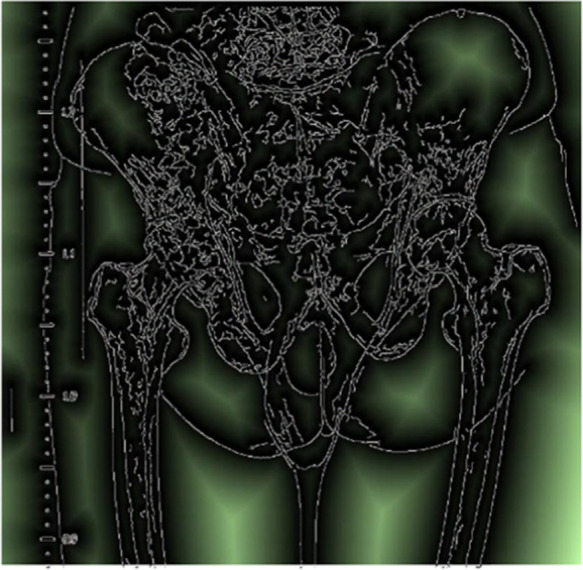
Bone edge registration, after edges were extracted using canny algorithm; and to determine the true bone edge, the bright areas are excluded as they are considered far from edges.

The closer the overlapping area is the better the two images match. The algorithm needs to traverse all the registration parameters and calculate the corresponding distance to find the smallest distance *A*, making A the smallest registration parameter; this study had taken it as the final registration result. Figure 2 illustrates how edges are registered after being detected.

#### X-ray image stitching

After determining the overlapping region, one of the most critical aspects of successful image stitching is how to combine the images, making this step crucial. Wavelet transformations are used in a wide range of applications, often replacing the Fourier Transform. Image compression, feature extraction, image denoising, and other medical image technologies benefit from Wavelet Transforms (WT) (11). Hence, this study used the wavelet transform domain stitching method to merge x-ray images together. Assuming that the overlapping areas of the two images were $I^{1},I^{2}$, and their wavelet transform coefficients were $W^{1},W^{2}$, respectively, the wavelet coefficients of the same scale were aligned:

$$W_{i}=\theta W_{i}^{1}+(1-\theta )W_{i}^{2}$$

**Figure 3:**
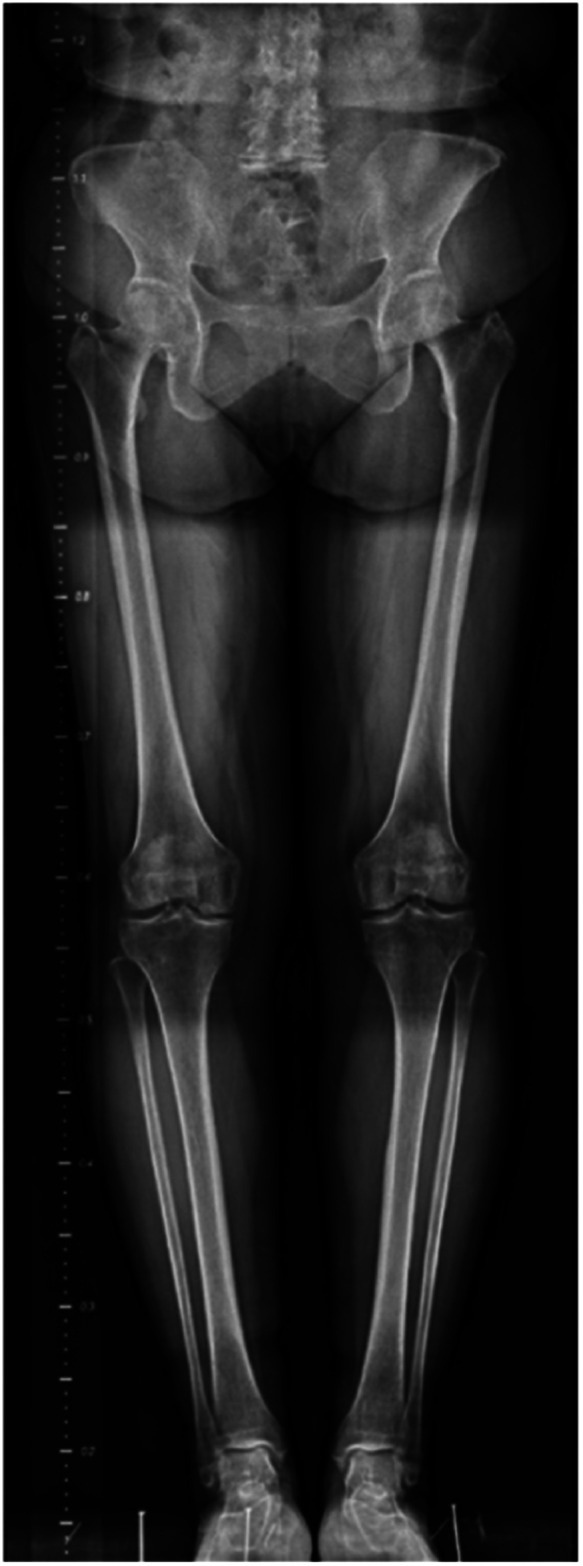
A full-length lower limb x-ray radiograph stitched using the proposed method based on canny algorithm.

After the aligning was completed, the wavelet transform was applied to produce the stitching result, as shown in Figure 3.

### HKA angle calculation

HKA angle is a commonly used angle in lower limb surgical interventions and planning (1, 12); therefore, we employ it to assess the quality of our stitching. The HKA angle calculations were performed manually and automatically.

In accordance with the criteria outlined in this book (12) for calculating the HKA angle, we first manually calculated the angle. Three distinct users were given the same set of x-ray images of the lower limb. Using an iPad application called Angle meter version 1.9, they estimated the midpoint of the femur, knee, and ankle and then calculated the HKA angle for each of the provided images. This app has a rating of 4.6 stars on the Apple store; it can measure angles from any image, but in this study, it was used to measure the HKA angle from the stitched x-ray images.

Second, the automated HKA angle calculation was performed on a Windows computer using Python and the RCNN algorithm (Region-Based Convolutional Neural Networks). RCNN employs selective search to identify candidate bounding-box object regions (a region of interest) (13). Each of these regions’ convolutional network options is then extracted for classification. Utilized in our research to automatically calculate the HKA for the purpose of the experiment.

### Analytics

We determined the *P*-value by performing a *T*-test on SPSS (Statistical Package for the Social Sciences) on all the HKA angle values obtained manually and automatically for the x-ray images stitched using this method and those constructed manually by an expert. A positive *P*-value (>0.05) indicates that there is no statistically significant difference between the HKA angles of the stitched x-ray images and those obtained by an expert. In addition, analysis of variance (ANOVA) and the *T*-test were used to examine the mean difference. Python, a conventional programming language, was used to execute the Canny algorithm and apply the steps of our method. This study utilized a Windows 10 computer with 16GB of RAM and an i7 processor.

## Results

### Manual HKA angle calculation outcomes

**Figure 4:**
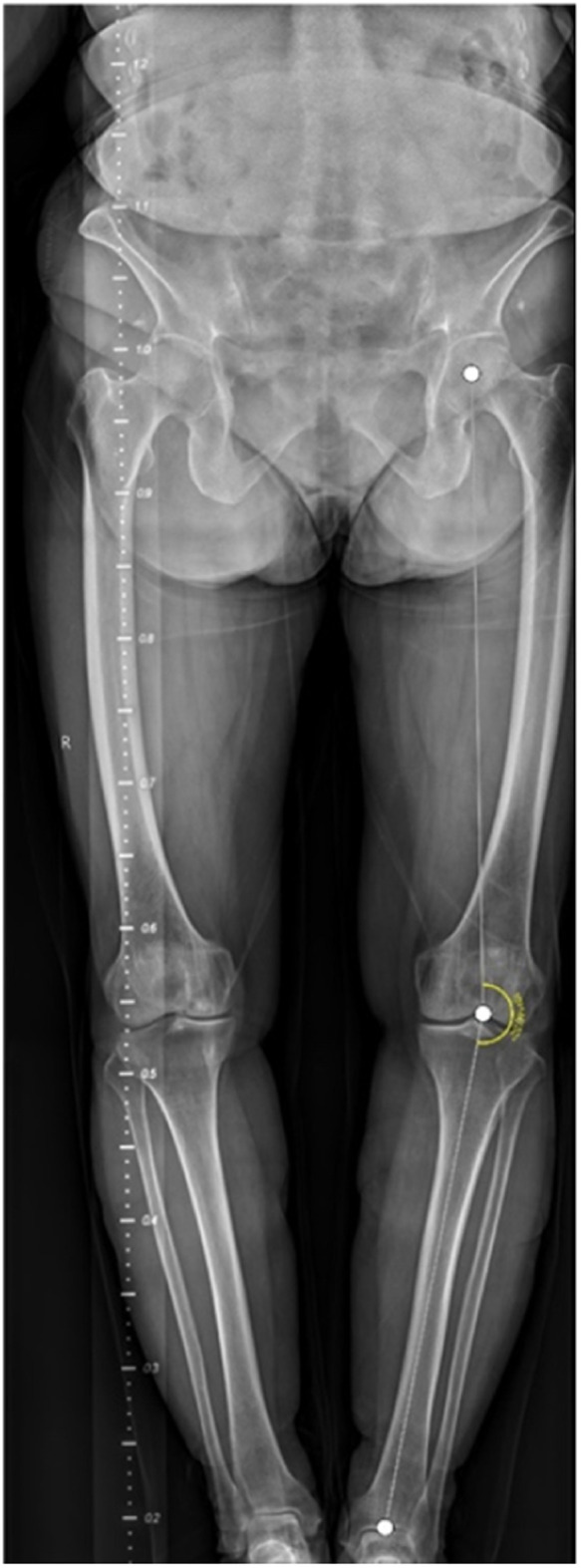
HKA angle calculated manually using angle meter. A mid-femur-head point is estimated, a mid-knee joint point and a mid-ankle joint point were estimated to get the HKA angle.

**Figure 5:**
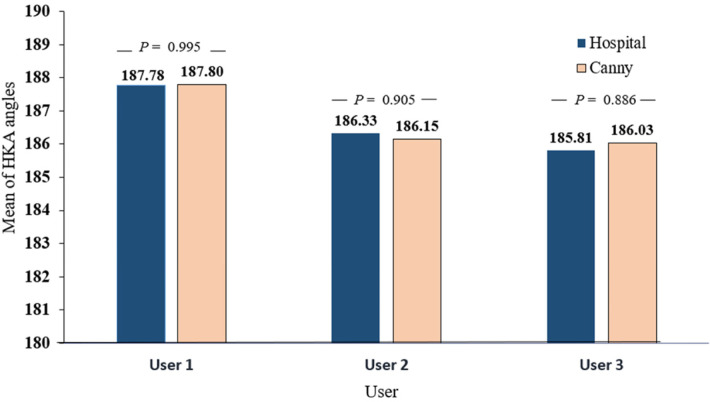
Manually measured HKA angles comparison for three different users between x-ray images stitched by an expert and acquired from the hospital and x-ray images stitched using our method based on canny algorithm.

Using the Angle Meter APP on an iPad, three separate users manually measured the HKA angle for both images stitched by an expert and others obtained using Canny; Figure 4 depicts an example of the angle measurement result. We then compared the mean HKA angles between all images stitched with this method and those stitched by an expert and found *P* = 0.995 for user 1, *P* = 0.905 for user 2, and *P* = 0.886 for user 3. Figure 5 shows the difference in means. We then used ANOVA on SPSS to compare the mean to the standard deviation between the three different users, as shown in Table 1.

**Table 1:** The mean compared to the standard deviation for both x-ray images obtained from the hospital and were constructed by an expert and the ones stitched using our method based on canny by three different users.

Variable	User 1 (*n* = 28)	User 2 (*n* = 28)	User 3 (*n* = 28)	*P-*value
Hospital, mean ± SD	187.78 ± 1.19	186.34 ± 1.14	185.81 ± 1.10	**0**.**010**
Canny, mean ± SD	187.80 ± 1.18	186.15 ± 1.13	186.03 ± 1.08	**0**.**020**

### Automated HKA angle calculation outcomes

**Figure 6:**
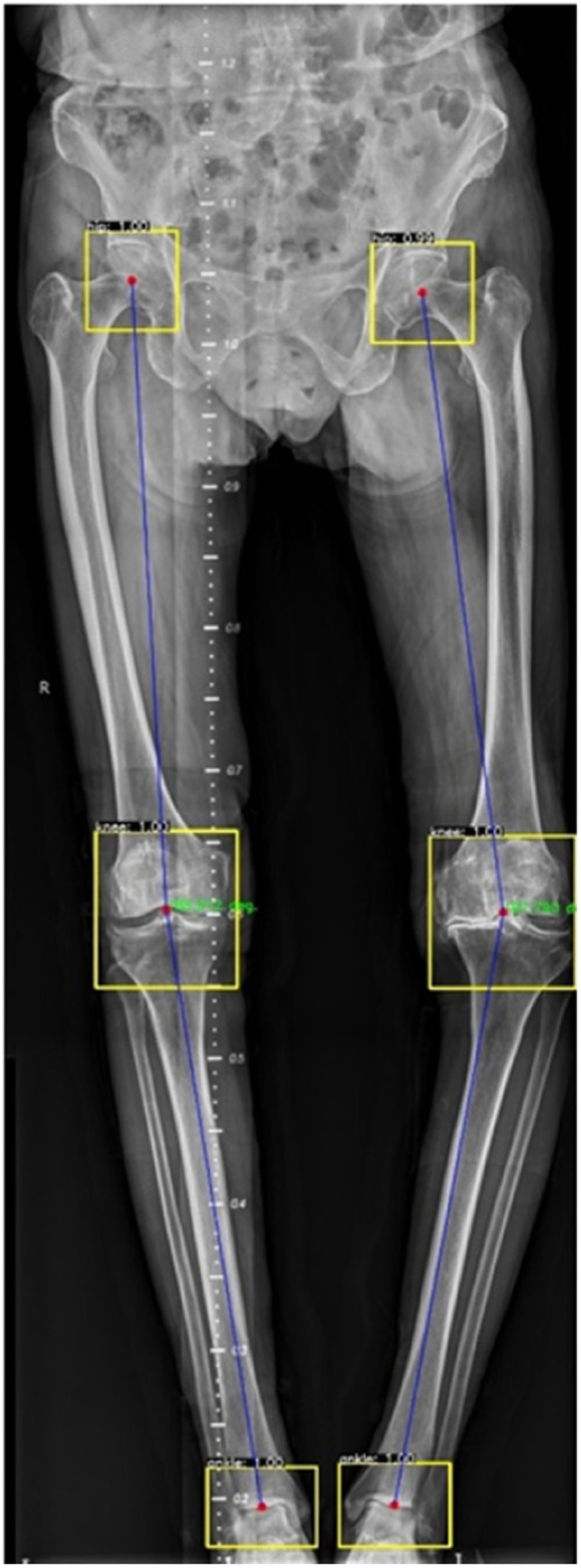
HKA angle calculated automatically using RCNN.

**Figure 7:**
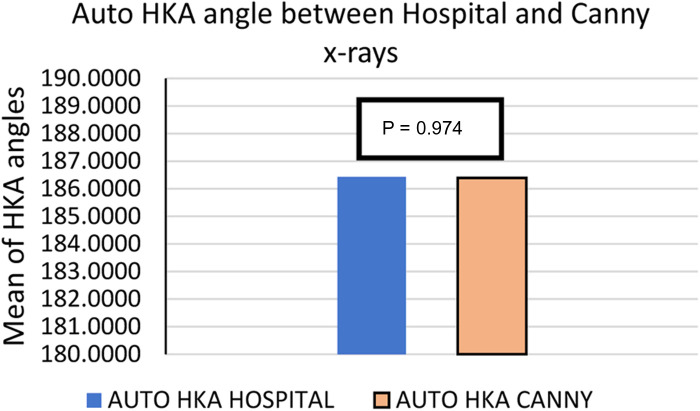
The mean of HKA angles for 28 sets of x-ray images stitched using our method and the x-ray radiographs obtained from the hospital directly, which were stitched by an expert.

On a Windows computer running Python and using RCNN, we automatically calculated the HKA angle for both sets of images obtained by Canny and stitched by an expert. An example of the result is displayed in Figure 6. After measuring the HKA angle automatically, we ran a *T*-test on SPSS to compare the means to the standard deviation and determined whether there was any significant difference or not. Hence, we found *P* = 0.974 as can be seen in Table 2 and Figure 7.

**Table 2:** The mean compared to the standard deviation for auto-calculated HKA angles using *t*-test for both images obtained from the hospital that were constructed by an expert and others stitched using our method based on canny.

Variable	Automatic (*n* = 28)	*P-* value
Hospital, mean ± SD	186.45 ± 1.09	0.974
Canny, mean ± SD	186.40 ± 1.09	** **

## Discussion

In this study, we propose a new stitching method for lower limb x-ray images that are both efficient and accurate and is based on the detection of anatomical features. To evaluate this method, we first stitched 28 sets of lower limb x-ray images obtained from the Second affiliated hospital of Xi’an Jiaotong University, and then measured the HKA angles both manually and automatically. We have used HKA angle as a reference point because its practical and it is what full-length x-rays are used for. Using the calculated angles, we tested the data with the *T*-test; when comparing the means, the *P*-value was positive (>0.05). And when the mean was compared to the standard deviation, the *P*-value was also positive (>0.05). Which indicates no significant difference between the stitched x-ray images and the ones manually constructed by an expert. As we are merely depending on bone edges to stitch images, only 3 s are required to successfully construct a full-length x-ray image using our method. In Table 3, we compared several previous stitching techniques pertaining to the same topic. In 2013, Samsudain presented a pixel-based method to stitch x-ray images automatically. His technique utilized the MACE filter to detect x-ray images’ edges and corners; then, it estimated the overlapping region between two images and performed stitching accordingly. This method was applied only on a pair of x-ray images of a hand, and it was found 80% accurate. In 2016, Yang implemented a correlation coefficient (CC) for x-ray image edge detection, which is also based on a pixel-based image stitching. His study was mainly focused on scoliosis patients and their x-ray images. Despite his fair accuracy rate of 90%, his method was not evaluated for lower limb x-ray stitching. In 2018, Ben Zekri applied a feature-based x-ray image stitching algorithm that used Sobel kernel and a second derivative filter that detected lower limb bone edges and estimated the bone shaft. This robust image stitching method took 15 s to create a panoramic x-ray image. His method was tested on a big database of lower limb x-ray images and was found to have a 100% accuracy rate. But we believe that using bone edges alone is both sufficient and fast to create a panoramic x-ray image of the lower limb as demonstrated in our study where we also obtained a 100% accuracy rate while reducing the time to three seconds only. In general we can say that Pixel-based stitching, despite being faster, it less accurate than feature-based method. Nonetheless, a key advantage of feature-based stitching is that it can stitch multiple x-ray images at once, in this case its three images that are required to construct a full-length radiograph of the lower limb. This method is more time-consuming, but it produces superior results.

**Table 3:** A comparison between different stitching methods and their efficacy.

Name	Year	Method	Images	Accuracy	Time	Rotation/scaling/translation
Samsudain	2013	Pixel-based method (MACE filter)	2 images of a hand were used	80%	2.2 s	No
Yang	2016	Pixel-based method (multiple correlation coefficient)	2 images of the spine were used	90%	1.9 s	Rotation, scaling and translation
Ben Zekri	2018	Feature-based method (sobel algorithm)	3 lower limb images, the hip, the knee, and the ankle	100%	15 s	Scaling and rotation
Tariq	2021	Feature-based method (canny algorithm)	3 lower limb images, the hip, the knee, and the ankle	100%	3 s	No
Manual (used by the hospital of this study)	N/A	Expert stitching manually	3 lower limb images, the hip, the knee, and the ankle	100%	90 s	Rotation, scaling and translation

**Figure 8:**
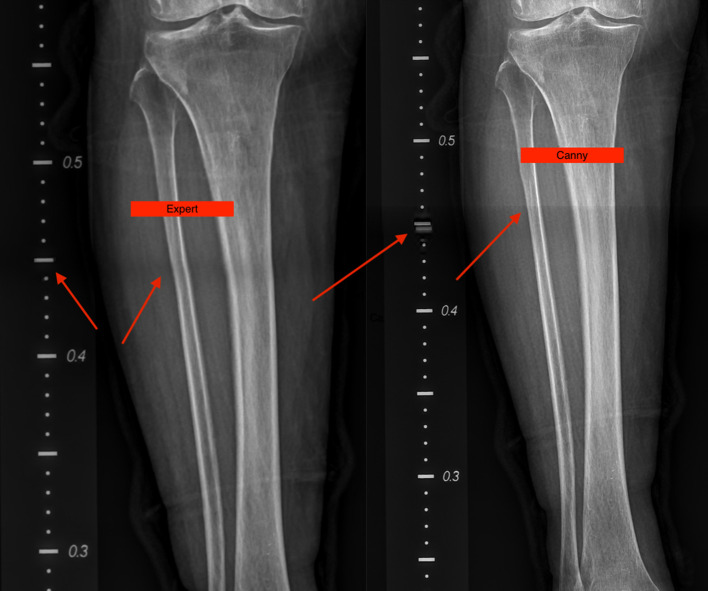
A slight distortion can be seen on the ruler’s markers in the image obtained by canny to the right while maintaining accurate anatomical features. Whereas, no distortion in the ruler markers’ can be seen in the image stitched by an expert to the left but a slight distortion in the anatomical features in present.

**Figure 9:**
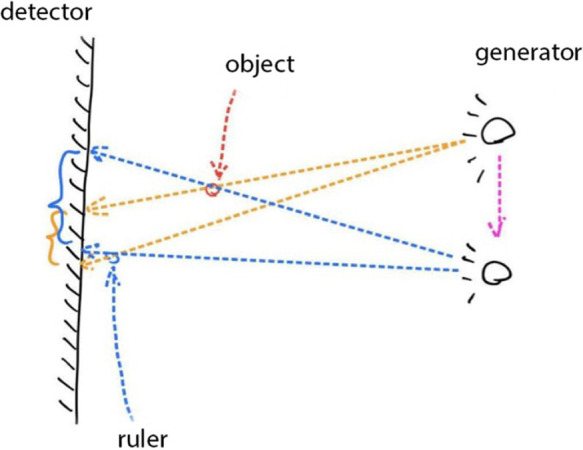
During the x-ray image capturing process, the source has to move from top to bottom to take multiple images; this means that the ruler markers’ is captured from two different angles. Once the images are stitched according to features and not ruler markers’, a slight distortion can be seen in the markers. It does not represent a real distortion in the stitching of our method, but it verifies that our method solely depends on anatomical features to perform stitching and that its more accurate.

In some of the stitched images using our method, we found a distortion in the ruler’s markings, as can be seen in Figure 8 to the right. Though, manual stitching depends on external markers as stated earlier, and in our hospital’s case, that marker is a ruler. But that is not always sufficient as seen in the image stitched by an expert to the left in Figure 8. As the expert depends mainly on the markers to perform his stitching, this could present some anatomical mismatching or slight distortion. On the other hand, our method solely uses anatomical features to perform the stitching with superior performance. This phenomen can be explained in Figure 9. As linear x-ray systems have the x-ray source moving vertically from top to bottom, and while the patient and the markers used are fixed at one position. The ruler is captured from different angles, thus, using it as the only factor to stitch x-ray radiographs could present such an anatomical distortion as in Figure 8 to the left. Whereas using our method, the findings don’t represent a distortion in the anatomical features, and that indeed further proves that our method is even more accurate when compared to this manual method.

This study has several limitations, including the inability to scale or rotate x-ray images prior to stitching. However, as previously explained, this is irrelevant because patients must remain still and immobile during the image capture process, eliminating the possibility of shifting or transitioning. Moreover, the tests were only conducted on x-ray images from the Second Affiliated Hospital of Xi’an Jiaotong University, so we do not know the effectiveness of this method on x-ray images from other sources.

## Conclusion

This study proposes a new method for stitching x-ray images of the lower limb. Our method reduces overall processing time while maintaining a high level of accuracy which is 100%. Using the Canny algorithm to detect bone edges that exist within the x-ray image itself makes this method extremely user-friendly and eliminates the need for additional markers or rulers. In conclusion, this method is a reliable technique for stitching x-ray images of the lower limb.

## Data Availability

The raw data supporting the conclusions of this article will be made available by the authors, without undue reservation.
